# Blood glucose levels and the risk of HPV multiple infections in high-grade squamous intraepithelial lesions: A retrospective cross-sectional study of Chinese patients

**DOI:** 10.1097/MD.0000000000030494

**Published:** 2022-09-16

**Authors:** Jie Zhou, Xiang Cai Wei, Hong Yan Xu, Hong Bo Hu, Fan Xiang Li, Wei Juan Zhou, Ye Chen, Zhen Liu

**Affiliations:** a Jinan University, Guangzhou, Guangdong, China; b Department of gynecology, Yuebei People’s Hospital, Shaoguan, Guangdong, China; c Department of Obstetrics and Gynecology, The First Affiliated Hospital, Sun Yat-sen University, Guangzhou, China.

**Keywords:** association, generalized additive model, glucose, HPV multiple infections

## Abstract

Besides the controversy of the association of high glycemic index and glycemic load with precancerous cervical lesions, only a few studies have examined the impact of fasting blood glucose levels on human papillomavirus (HPV) multiple infections. In the present study, we appraised the relationship between blood glucose levels and multiple HPV infections in a population of HPV-positive women with cervical high-grade squamous intraepithelial lesions (HSIL). The present study was designed as a cross-sectional correlative analysis. A total of 560 participants with a pathologically confirmed HSIL with HPV infection were included from a hospital in China during January 1, 2018, and December 31, 2019. The target variables and the outcome variables were the glucose levels at the baseline and HPV multiplicity, respectively. The odds ratio and 95% confidence intervals were calculated to estimate the risk of multiple infections via logistic regression analysis. The average age of the 560 participants was 44.63 ± 10.61 years; the nonlinear relationship was detected between the glucose levels and multiplicity of HPV, with an inflection point at 5.4. After adjusting for the full range of variables, the effect sizes and confidence intervals for the left and right sides of the inflection points were found to be 0.379 (0.196–0.732) and 5.083 (1.592–16.229), respectively. In this cross-sectional study, both high and low blood glucose levels increased the risk of multiple HPV infections, demonstrating a U-shaped relationship between the blood glucose levels and multiple HPV infections.

## 1. Introduction

Cervical cancer is the fourth leading cause of cancer in women worldwide, posing a great threat to the entire world population, especially the developing countries.^[1]^ Persistent human papillomavirus (HPV) infection is widely known as the etiological cause of cervical intraepithelial neoplasia (CIN) and cervical cancer.^[2,3]^ Past studies have demonstrated that the development of cervical cancer takes years to even decades, including the intermediate stage of CIN.^[4]^ Some studies have reported that multiple HPV infections contributes to the growing risk of cytological abnormalities, when compared to a single HPV infection,^[5]^ and hence, it is considered as a risk factor for cervical cancer development.^[6–8]^ Cervical infections with more than one HPV genotype are common in young women, and approximately 25% to 49% of the affected women have been found to have multiple infections.^[9–11]^ More than 200 types of HPV have been isolated so far, of which 12 to 15 form the high-risk types.^[12,13]^ High-risk HPV is the leading cancer-causing type, HPV-16 and -18 are the main carcinogenic HPV, and other high-risk types include HPV-31, -33, -35, -45, -52, and -58.^[14]^ In addition, the type and pattern of HPV infection vary by geography and economy.^[15,16]^

Past studies have reported that glucose transporters (GLUTs) are associated with the malignant transition of cervical epithelium with HPV infection.^[17]^ In HPV-positive cervical cancer, the expression of GLUT1 indicates a worse prognosis.^[18]^ Oncoproteins E6 and E7 expressed by HPV play critical roles in cervical carcinogenesis.^[19]^ Jia Yi Tang et al reported that HPV E6/E7 upregulated GLUTs in lung cancer and that E6 and E7 mediated GLUT expression and glucose absorption were related to lung cancer.^[20]^ Therefore, we inferred that HPV E6/E7 upregulates glucose E6, E7-mediated GLUT expression, and glucose absorption, which lead to the malignant transition of the cervical epithelium with HPV infection. Conclusively, GLUT1 is a promising diagnostic approach, wherein the glucose metabolism can potentially act as an intervention target in the treatment of cervical cancer.^[17]^

Glucose, as the vital energy resource in cells, is extremely common in the human blood.^[21]^ Numerous studies have indicated a strong association between increased levels of glucose and adverse health outcomes, including hypertensive diseases, metabolic disturbance, kidney diseases, heart diseases, and tumor.^[22–24]^ However, according to the current studies, the relationship between glucose and cancer is inconsistent.^[25]^ Several epidemiological studies and meta-analyses have examined the association among glycemic index (GI), glycemic load (GL), and the risk of some cancers, such as ovarian cancer,^[26,27]^ endometrial cancer,^[28,29]^ breast cancer,^[30,31]^ liver cancer,^[32,33]^ colon cancer,^[34,35]^ bladder cancer,^[36]^ and renal cancer.^[37]^ However, the results of these studies are conflicting.^[25]^ Some studies have demonstrated a strong correlation between dietary GI or GL and cancer risk,^[25,30,37,38]^ whereas others have reported no such association.^[29,33,35,39]^ Navarro-Meza et al reported that high levels of glucose in the plasma and obesity can act as risk cofactors in the development of precancerous lesions of the cervix.^[40]^ Similarly, in a Korean case-control study on 1340 cases (670 controls and 262, 187, and 221 patients with CIN1, CIN2/3, and cervical cancer, respectively), GL and GI were identified to be positively associated with increased risk of CIN1, but not with the risk of CIN2/3 and cervical cancer.^[17]^

However, no previous epidemiological studies have reported any relationship between glucose and the risk of multiple HPV infections. One study reported a strong relationship between a high level of blood glucose and a high risk of cervical cancer with a poor prognosis.^[40]^ The present study aimed to evaluate the relationship between glucose levels and the risk of HPV infection among Chinese women with pathologically confirmed HSIL.

## 2. Participants and Methods

### 2.1. Study design

In this research, a retrospective study was conducted to explore the relationship between glucose and multiple HPV infections. The independent variable was the glucose level obtained at the baseline. The dependent variable was the type of HPV infection.

### 2.2. Study population

The data of participants were gathered from the electronic medical records system of the Gynecology Department, Yuebei People’s Hospital, Shaoguan City, China. To safeguard patient privacy, no personally identifiable enrollment data were included. The data were obtained from the hospital’s electronic medical record system. Informed consent from participants was waived off as this was a retrospective cohort study. The Hospital Institutional Review Board approved the study protocol.

**Figure 1.**
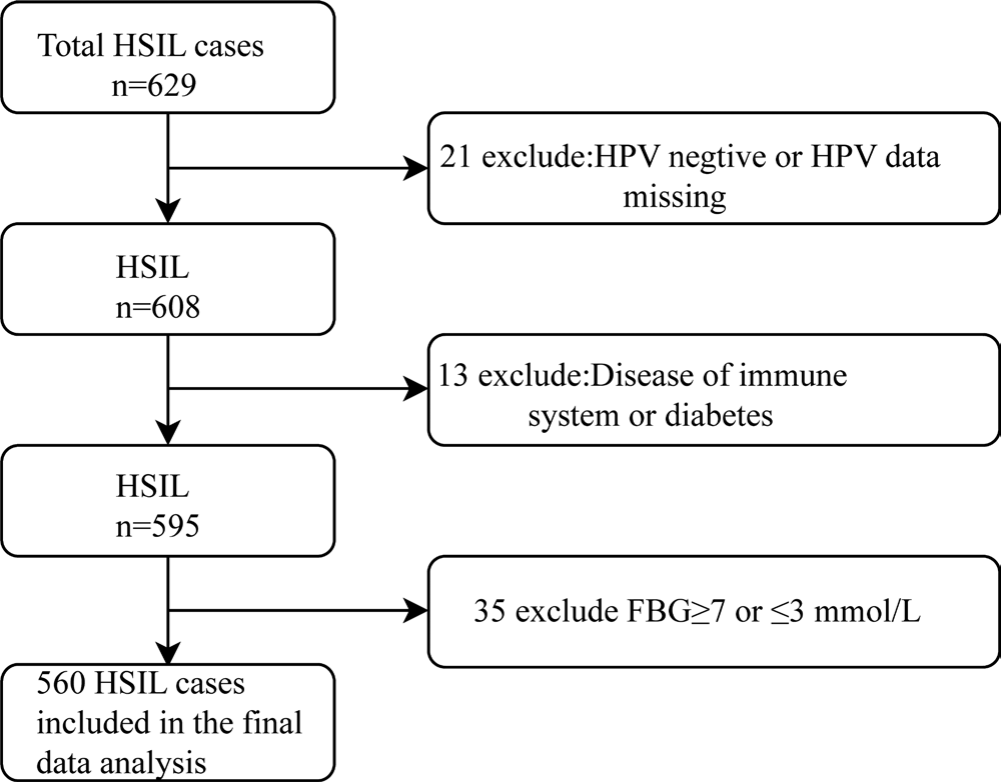
Composition of enrolled patients’ population. FBG = fasting blood glucose, HSIL = high-grade squamous intraepithelial lesion, HPV = human papillomavirus.

Figure 1 illustrates a flow chart of the participant enrollment process. A retrospective analysis was undertaken of 629 patients diagnosed with HSIL who then underwent colposcopy-guided biopsy (CGB) at the Yuebei People’s Hospital between January 2018 and December 2019. We enrolled patients for whom the HPV genotype information was available before CGB. Of these 629 patients, 21 were excluded due to presurgical hrHPV negativity or the lack of HPV data. Thirteen patients were excluded because they had acquired or congenital immunosuppression or diabetes or were undergoing chronic immunosuppressive treatments. Patients with fasting time of < 8 hours and the blood glucose level of ≥ 7 and ≤ 3 were also excluded. Finally, a total of 560 patients were recruited for this study.

## 3. Variables

### 3.1. Glucose testing

We acquired the glucose level at the baseline and recorded it as a continuous variable. The procedure was as follows: blood samples were collected in EDTA-anticoagulated tubes and subjected to biochemistry tests the next morning after the patients were admitted. The blood samples collection and analysis of the fasting blood specimens were performed after a minimum of 8 hours of overnight fast to define the fasting glycemia. According to the existing research, we obtained the outcome variable (dichotomous variable).

### 3.2. HPV genotyping

The detailed process of people who were infected with HPV was described as follows: infection types were “any HPV infection” and “multiple HPV infection” (2 or more genotypes). HPV genotyping was performed on specimens collected by using the HPV typing test kit (PCR + membrane hybridization) analysis. A Chinese FDA approval was obtained for this test (approval number [2014]: 3402188).

HPV genotypes were identified using the YanengBio Human Papillomavirus Genotyping Kit (YanengBio, Inc., Shenzhen, China) according to the manufacturer’s instructions. The 15 HPV types include 13 high-risk types (i.e., HPV-16, HPV-18, HPV-31, HPV-33, HPV-35, HPV-39, HPV-45, HPV-51, HPV-52, HPV-56, HPV-58, HPV-59, and HPV-68 detected) and 2 low-risk types (i.e., HPV-6 and HPV-11). According to the information provided by YanengBio Human Papillomavirus Genotyping Kit (YanengBio, Inc.), the parameter for amplification was set to the following: 95°C for 9 minutes, 40 cycles (95°C for 20 s, 55°C for 30 s, 72°C for 30 s), 72°C for 5 minutes. After amplification, hybridization, and color development, positive test results were noted as a blue spot. Positive dots were identified according to the distribution of the HPV types in the film strips. Both negative and positive controls were set up throughout. The experiment was subjected to internal quality control and external quality assessment, and the results were found to meet the requirements.

The covariates employed in this study fell into the following categories: population demographics; variations that affected the blood glucose levels or HPV multiple infections that had been previously reported in the literature or were based on our clinical experience. Hence, totally adjusted models were constructed using the following variables: continuous variables: age (year), weight (kg), neuron-specific enolase (NSE = µg/mL), squamous cell carcinoma of the anus (SCCA = ng/mL), alanine aminotransferase (ALT = U/L); aspartate aminotransferase (AST = U/L); uric acid (UA = Ummol/L); glucose (GLU = mmol/L); creatinine (CREA = Ummol/L); urea nitrogen (BUN = mmol/L) (obtained at the baseline); categorical variables: vagina cleanness (I–II was defined as normal and III–IV as abnormal), menopausal status (premenopause or postmenopause) was defined as no menstrual period for the past 12 months. Several pregnancies (0, 1–3, ≥4), the number of childbirth (unfed females or 1 primiparity [one delivery], multiparous [2 delivery], and grand multiparous [≥3 deliveries]). Several miscarriages (0 denotes no such event and 1 + denotes 1 or more events). Cytology samples were interpreted by a pathologist with reference to the Bethesda System.^[2]^ The cytology samples were interpreted by a pathologist following the Bethesda SystemNILM or ASC-US code with 1, A SC-H or HSIL or LSIL code with 2). A histological diagnosis of the excised specimen was based on the World Health Organization specified classification system.

### 3.3. Statistical analysis

In this study, our representations of the continuous variables were based predominantly on whether they are normally distributed. If they were normally distributed, we expressed continuous variables as the mean ± standard or median and conversely as median (min, max). The categorical variables were then expressed as frequencies or percentages. We used χ2 (categorical variables), a 1-way ANOVA test (normal distribution), or Kruskal–Wallis H-test (skewed distribution) to test for a difference between different multiples of HPV groups (dichotomous). The process of analyzing the entire data was separated into 3 steps. Step 1: Using univariate and multivariate binary logistic regression analyses. A total of 3 models were constructed: Model 1, with no adjustment for covariates; Model 2, adjusted for socio-demographic data only; Model 3, Model 2 + other coefficients are shown in Table 1 (end of article). Step 2: A generalized additive model and smoothed curve fitting (the penalized spline method) were performed to address the nonlinearity of glucose and multiple infections of HPV. If nonlinearity was detectable, we first computed the point of inflection using a recursive algorithm and then built a 2-segment binary logistic regression on either side of the point of inflection. We established the best fitting model based on the *P* value of the log-likelihood ratio test. Step 3: Subgroup analyses were undertaken by employing a hierarchical binary logistic regression model. For continuous variables, we first transformed them into categorical variables based on the clinical cut points or trisections, followed by interaction tests. The likelihood ratio tests were performed after correcting for the effects of subgroup indicators. To ensure that the data analysis was robust, a sensitivity analysis was performed. We switched glucose to a trichotomous variable and counted the *P* for trend. The study aim was to validate the results for glucose as a continuous variable and to determine the possibilities for nonlinearity. All analyses were performed using the statistical software packages R (www.r-project.org) and Empower Stats (http://www.empower stats.com; X&Y Solutions, Inc., Boston, MA). Significant *P* values < .05 (both the sides) were deemed to be statistically significant.

**Table 1:** Baseline characteristics of participants.

Characteristics*	Single HPV infection (n = 375)	Multitype HPV infection (n = 153)	*P* value
Age, y, median (IQR)	44.520 ± 10.077	43.843 ± 11.514	.502
Vagina cleanness, n (%)			.688
I–III grade	355 (95.430%)	140 (94.595%)	
IV grade	17 (4.570%)	8 (5.405%)	
Vaginal leukocytes, n (%)			.192
+–+++	320 (85.791%)	121 (81.208%)	
++++	53 (14.209%)	28 (18.792%)	
Menopausal status, n (%)			.565
Premenopause	253 (71.875%)	104 (69.333%)	
Postmenopause	99 (28.125%)	46 (30.667%)	
Number of pregnancies, n (%)			.047
0	10 (2.695%)	8 (5.229%)	
1–3	222 (59.838%)	75 (49.020%)	
≥4	139 (37.466%)	70 (45.752%)	
Number of childbirth, n (%)			.549
0	77 (20.924%)	26 (16.993%)	
1–2	207 (56.250%)	88 (57.516%)	
≥3	84 (22.826%)	39 (25.490%)	
Number of miscarriages, n (%)			.526
3≤	346 (94.278%)	142 (92.810%)	
≥4	21 (5.722%)	11 (7.190%)	
Tct, n (%)			.471
ASC-US≤	120 (39.735%)	51 (43.590%)	
≥LSIL	182 (60.265%)	66 (56.410%)	
Weight, median (IQR)	55.261 ± 9.250	55.082 ± 7.817	.833
NSE, median (IQR)	12.565 ± 3.347	13.114 ± 3.709	.129
SCCA, median (IQR)	0.484 ± 0.944	0.419 ± 0.205	.42
ALT, median (IQR)	18.463 ± 9.627	18.929 ± 11.312	.632
AST, median (IQR)	18.425 ± 6.164	18.661 ± 6.760	.698
GLU, median (IQR)	5.017 ± 0.594	4.906 ± 0.678	.068
UA, median (IQR)	286.877 ± 67.695	299.448 ± 64.100	.05
CREA, median (IQR)	62.517 ± 10.582	62.337 ± 10.127	.858
BUN, median (IQR)	4.463 ± 1.100	4.401 ± 1.184	.562

ALT = alanine aminotransferase, AST = aspartate aminotransferase, BUN = urea nitrogen, CREA = creatinine, GLU = glucose, IQR = interquartile range, NSE = neuron-specific enolase, SCCA = neuron-specific enolase, UA = uric acid.

## 4. Results

### 4.1. Characteristics of the selected participants at the baseline

Altogether 560 participants were selected for analysis of the final database after screening using inclusion and exclusion criteria (see Fig. 1 for flow chart). We have shown the baseline characteristics of these selected participants in Table 1, which were based on a dichotomy of whether they were multiply infected with HPV. Overall, the mean age of the 560 selected participants was 44.63 ± 10.61 years. There were no statistically significant differences detectable for age, weight, NES, SCCA, ALT, AST, UA, CREA, BUN, glucose, vagina cleanness, vaginal leucocytes, menopausal status, the number of childbirth, the number of miscarriages, and TCT among different multiplicity of HPV groups, but there were differences in the number of pregnancies (*P = .047*).

### 4.2. Univariate analysis

We have presented the outcomes of the univariate analysis in Table 2. Using univariate binary logistic regression, we observed that glucose was not associated with the diversity of HPV. However, we found that high glucose tertiles (0.49, 0.31–0.77 vs ref) were adversely associated with the multiplicity of HPV.

**Table 2:** Number of HPV infection and related factors in single-factor analysis.

Number of HPV infection	OR (95% CI)	*P* value
Age, y	0.99 (0.98–1.01)	.527
Vagina cleanness	1.43 (0.64–3.19)	.386
Vaginal leukocytes	1.44 (0.89–2.33)	.134
Menopausal status	1.11 (0.74–1.66)	.607
Number of pregnancies	0.43 (0.17–1.08)	.074
Number of childbirth	1.53 (0.87–2.67)	.138
Number of miscarriages	1.31 (0.63–2.74)	.466
Tct	0.87 (0.57–1.33)	.529
Weight	1 (0.98–1.02)	.909
NSE	1.04 (0.98–1.1)	.164
SCCA	0.79 (0.41–1.52)	.476
ALT	1 (0.98–1.02)	.829
AST	1 (0.97–1.03)	.945
Glu	0.79 (0.59–1.07)	.126
UA	1 (1–1)	.114
CREA	0.99 (0.97–1.01)	.354
BUN	0.99 (0.84–1.16)	.904
Glutritertile 1	1	
Glutritertile 2	0.66 (0.42–1.03)	.067
Glutritertile 3	0.49 (0.31–0.77)	.002

95% CI = 95% confidence interval, ALT = alanine aminotransferase, AST = aspartate aminotransferase, BUN = urea nitrogen, CREA = creatinine, GLU = glucose, HPV = human papillomavirus, NSE = neuron-specific enolase, OR = odds ratio, SCCA = neuron-specific enolase, UA = uric acid.

### 4.3. Results of unadjusted and adjusted binary logistic regression

In this study, 3 models were constructed to analyze the independent effect of glucose on HPV multiple infections (univariate and multivariate binary logistic regression analyses). The effects sizes (odds ratio) and 95% confidence intervals (CI) are presented in Table 3. In the nonadjusted model (model 1), the effect size based on the model was interpreted as the difference between 1 unit of glucose and the risk of multiple effect events. For instance, an unadjusted model with an effect size of 0.75 for the risk of multiple HPV infections indicated that a 1-unit difference in glucose was associated with a 25% difference in the risk of multiple effect events (0.75, 95% CI 0.58–0.98). In the minimum Adjusted Model (Model 2), a 1 unit change in glucose was associated with a 19% increase in the risk of multiple effect events (0.81, 95% CI 0.59–1.11). Within the fully adjusted model (Model 3) (adjustment for all covariates listed in Table 1), for each additional 1 unit of glucose, the risk of an event of multiple effection increased with a decrease of 16% (0.84, 95% CI 0.57–1.22).

**Table 3:** The results of univariate and multivariate analyses.

	Nonadjusted model OR (95% CI)	Minimally adjusted model OR (95% CI)	Fully adjusted model OR (95% CI)
Glu	0.75 (0.58–0.98)	0.81 (0.59–1.11)	0.84 (0.57–1.22)
Glu (tritertile)			
Q1	Ref	Ref	Ref
Q2	0.73 (0.49~–1.07)	0.69 (0.46–1.02)	0.78 (0.45–1.33)
Q3	0.45 (0.3–0.68)	0.43 (0.28–0.65)	0.48 (0.27–0.84)
*P* for trend	<.001	<.001	.01

Nonadjusted model: we did not adjust any covariate; Minimally adjusted model: we only adjusted age, and weight; Fully adjusted model: we adjusted age, vagina cleanness, vaginal leukocytes, menopausal status, number of pregnancies, number of childbirth, number of miscarriages, Tct, weight, NSE, SCCA, ALT, AST, UA, CREA, BUN.

95% CI = 95% confidence interval, ALT = alanine aminotransferase, AST = aspartate aminotransferase, BUN = urea nitrogen, CREA = creatinine, GLU = glucose, HPV = human papillomavirus, NSE = neuron-specific enolase, OR = odds ratio, SCCA = neuron-specific enolase, UA = uric acid.

We switched glucose from a continuous variable to a categorical variable (triple quantile of glucose) for sensitivity analysis, and the *P* for the trend in glucose in the fully adjusted model with a categorical variable was inconsistent with the results when glucose was a continuous variable (Table 3). In addition, we found that the trends in effect sizes were nonequally spaced across the glucose groups.

### 4.4. The results of nonlinearity of glucose and multiplicity of HPV

**Figure 2.**
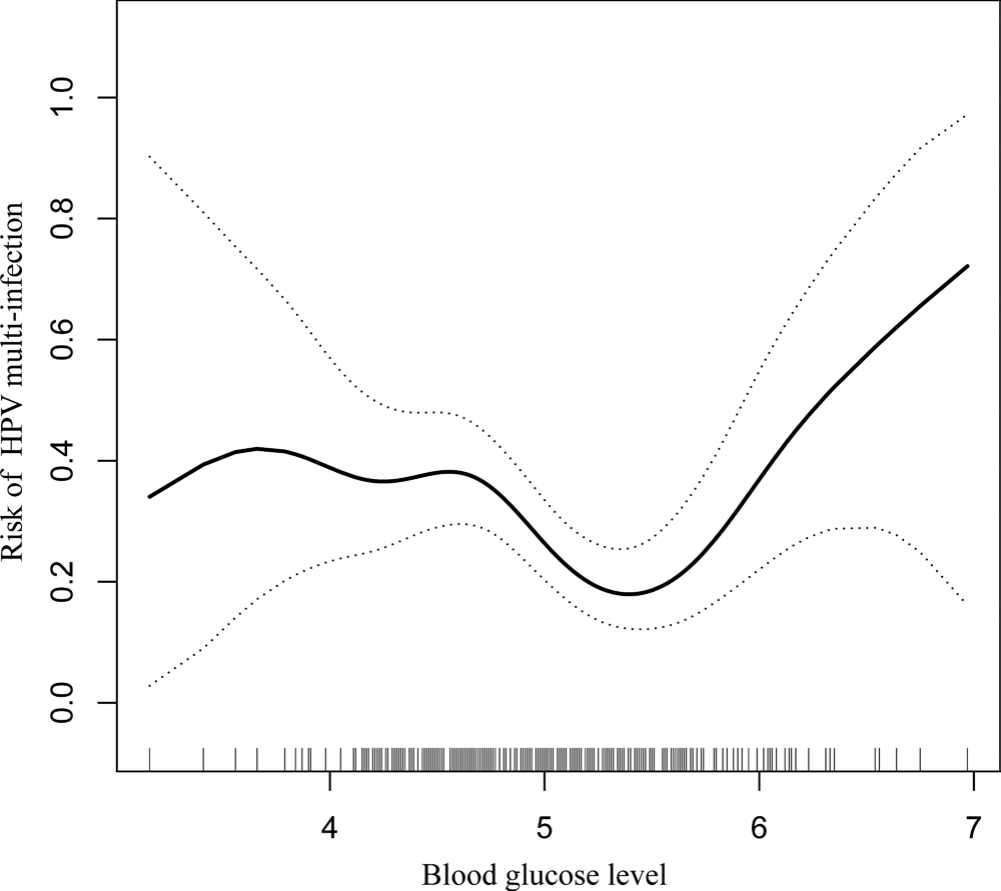
The relationship between the number of HPV infection and glucose level. A nonlinear relationship between them was detected after adjusting for age, vagina cleanness, vaginal leukocytes, menopausal status, number of pregnancies, number of childbirth, number of miscarriages, Tct, weight, NSE, SCCA, ALT, AST, UA, CREA, BUN. ALT = alanine aminotransferase, AST = aspartate aminotransferase, BUN = urea nitrogen, CREA = creatinine, HPV = human papillomavirus, NSE = neuron-specific enolase, SCCA = neuron-specific enolase, UA = uric acid.

In the present study, we investigated the nonlinear relationship between glucose and multiple HPV infections (Table 4, Fig. 2). The results of the smoothing curve and the generalized additive model demonstrated a nonlinear association with glucose levels and blood glucose levels after adjusting for age, weight, NES, SCCA, ALT, AST, UA, CREA, BUN, vagina cleanness (I–II was defined as normal, and III–IV as abnormal), vaginal leucocytes, menopausal status (premenopause or postmenopause), number of pregnancies (0, 1–3, ≥4), the number of childbirth (unfed females or 1 primiparity [1 delivery], multiparous [2 delivery], and grand multiparous [≥ 3 deliveries] and code with 0, 1, and 2), the number of miscarriages (0 denotes no event occurring and 1 + denotes one or more event), liquid-based cytologic test (TCT) (NILM or ASC-US code with 1, ASC-H or HSIL or LSIL code with 2). Both binary logistic regression and 2-segment binary logistic regression were applied to fit the association and the best-fit model based on *P* was selected for the log-likelihood ratio test.

**Table 4:** The results of 2-piecewise linear model.

	Multiple HPV infections (OR, 95% CI)
Fitting model by standard linear regression	0.841 (0.534, 1.323)
Fitting model by 2-piecewise linear regression	
Inflection point of glucose	5.39
<5.39	0.379 (0.196, 0.732)
>5.39	5.083 (1.592, 16.229)
*P* for log likelihood ratio test	.001

We adjusted we adjusted age, vagina cleanness, vaginal leukocytes, menopausal status, number of pregnancies, number of childbirth, number of miscarriages, Tct, weight, NSE, SCCA, ALT, AST, UA, CREA, and BUN.

95% CI = 95% confidence interval, ALT = alanine aminotransferase, AST = aspartate aminotransferase, BUN = urea nitrogen, CREA = creatinine, GLU = glucose, HPV = human papillomavirus, NSE = neuron-specific enolase, OR = odds ratio, SCCA = neuron-specific enolase, UA = uric acid.

As the *P* for the log-likelihood ratio test was < .05, we selected dichotomous binary logistic regression to fit the association between glucose and HPV multiplicity as it could accurately represent the relationship. Using a 2-segment binary logistic regression and recursive algorithm, we computed an inflection point of 5.39. On the left-hand side of the inflection point, the effect size and 95% CI were 0.379 (0.196–0.732), respectively. On the right-hand side of the inflection point, the effect size and 95% CI were 5.083 (1.592–16.229), respectively (Table 4).

## 5. Discussion

In this retrospective study on Chinese women infected with HPV, both hypoglycemia and hyperglycemia were significantly associated with an increased risk of multiple HPV infections. After further adjusting for the potentially confounding factors, including age, this association remained statistically significant. Moreover, the trend of the effect size was discontinuous on the left and right sides, respectively [Left 0.511 (0.360, 0.723); right 3.249 (1.375, 7.676)]. This result implied the presence of an independent U-shaped association between the glucose levels and multiple HPV infections.

To the best of our knowledge, it is imperative that a U-shaped curve has been used to describe the relationship between fasting glucose levels and the risk of HPV multi-infection, and these data can be applied to ascertain that the risk of multi-infection was the lowest in women with a high-grade cervical abnormality without diabetes at the glucose level 5.37 to 5.92 mmol/L. This information can facilitate the application of evidence-based quality improvement measures aimed at reducing the risk of multi-infection by maintaining the glucose level within a defined range of practices so as to reduce the risk of multiple infections.

Although the mechanisms linking the blood glucose levels to increased risk of HPV multiple infections are unclear, our research revealed that patients with a low blood sugar level are at a higher risk of multiple HPV infections. Moreover, hypoglycemia may be a manifestation of malnutrition, metabolic disorders, and poor immunity, while normoglycemia may represent more reliable immunity. These are some of the possible explanations. A past study showed that β-glucans have immunogenic and antiviral properties.^[41]^ β-glucan is a homogeneous polysaccharide composed of glucose as a monosaccharide and abundantly found in the cell walls of yeast, bacteria, and fungi. They consist of glucose molecules connected by (1-3), (1-4), or (1-6) beta-glycosidic links, have different branches structured separately from the linear trunk, and are known to mediate immunogenicity via receptor-mediated interaction with M cells.^[42–44]^ In addition to being immunogenic, it has a promising antiviral effect. For example, Jung et al demonstrated that the pulmonary lesions caused by infection with SIV were significantly more serious in animals predosed with β-glucan than those due to infection in the non-oral group. Moreover, there was a marked improvement in the levels of interferon-gamma (IFN-γ) and nitric oxide in the bronchoalveolar lavage liquid of the treatment group. These discoveries support the potential use of beta-glucan as a prophylactic/therapeutic alternative for flu virus infections.^[45]^ These results may cumulatively indicate that low blood sugar level is not good for the body to clear viruses.

In our study, subjects in the high glucose level group displayed a statistically significantly higher risk of acquiring HPV multiple infections. Hyperglycemia is associated with increased susceptibility to viral infection and cell-mediated immunodeficiency,^[46,47]^ which may make HPV clearance difficult, thereby leading to the promotion of cancer progression. Another study indicated that alterations in the insulin signaling pathways that promote glucose uptake and cell proliferation are frequently detected in cervical cancer.^[48]^ Positron emission tomography reveals that cancer constitutes more glucose than the surrounding normal tissues.^[49]^ Furthermore, mass spectrometry analysis revealed that glucose molecules, in addition to being an important source of energy, provides the building blocks required for cell replication, which explains the increased glucose metabolism in cancer cells and the outcomes of PET scanning.^[50]^ Upon viral infection, cells also experience increased glucose uptake to meet the substrate requirements for greater virus production. The increase in glycolytic metabolites and enzymes following adenovirus infection of the cells was found by Vander Heiden et al.^[50]^ Early on, Landini et al reported increased glucose uptake and metabolism in cells infected with cytomegalovirus^[51]^ as well as in cells infected with other subsequent viruses.^[52–54]^ Xie et al revealed that hyperglycemia leads to more severe coronavirus disease 2019 infections and poor prognosis.^[55]^ Thus, increased glucose uptake is a commonly observed feature of virally infected cells. Multiple studies have demonstrated that multiple infection patterns may be related to geographic, demographic, and socioeconomic factors.^[56,57]^

This study has the following clinical merits. To the best of our knowledge, this is the first time that an independent link between glucose and multiplicity of HPV in Chinese patients of a pathologically confirmed HSIL with HPV affection; and the outcome of this research should contribute to future studies in constructing models for the diagnosis or prediction of HPV multiplicity.

There are several strengths to our study. We addressed the issue of nonlinearity in this study and explored it further. This was an observational study and therefore prone to potential confounding influences. We minimized any residual confounding factors through rigorous statistical adjustments. We dealt with the targeted independent variables as constant and classified variables. This approach decreases the chance of data analysis and enhances the robustness of the results. There are some limits to this study, including the following: Chinese female patients with pathologically confirmed HSIL showing the HPV effect were selected as the study participants. Consequently, there were some inadequacies in the universalization and generalization of the study. As we eliminated HPV negative or missing HPV data, disease of the immune system, or diabetes, FBG ≥ 7 or ≤ 3 mmol/L, the outcome of this study does not apply to these individuals.

## 6. Conclusion

The blood glucose levels are associated with the risk of multiple HPV infections. High or low blood sugar level may increase the risk of multiple HPV infections, and the possible induction of TRIM by β-glucan can elicit protective effects by altering the immune response against a range of viral infections. Viral infection of cells also requires increased glucose uptake for viral replication and production. However, further research into the mechanisms of this problem is warranted. The assessment of glycemic management as an intervention is also recommended.

## Author contributions

X.C.W. presented the preliminary idea for this study. J.Z. was involved in initial draft writing. H.X., H.H. responsible for data analytics and discussion. F.X.L., Z.L., W.J.Z. gathering and cleaning data. Y.C. advice on revising articles.

## Acknowledgments

The authors acknowledge the patients.
